# Prevalence of Depression Among Empty-Nest Elderly in China: A Meta-Analysis of Observational Studies

**DOI:** 10.3389/fpsyt.2020.00608

**Published:** 2020-07-07

**Authors:** Hong-He Zhang, Yuan-Yuan Jiang, Wen-Wang Rao, Qing-E Zhang, Ming-Zhao Qin, Chee H. Ng, Gabor S. Ungvari, Yu-Tao Xiang

**Affiliations:** ^1^Department of Psychiatry, Xiamen Xianyue Hospital, Xiamen, China; ^2^Unit of Psychiatry, Faculty of Health Sciences, Institute of Translational Medicine, University of Macau, Macao, China; ^3^Center for Cognition and Brain Sciences, University of Macau, Macao, China; ^4^The National Clinical Research Center for Mental Disorders & Beijing Key Laboratory of Mental Disorders Beijing Anding Hospital & the Advanced Innovation Center for Human Brain Protection, Capital Medical University, School of Mental Health, Beijing, China; ^5^Department of Geriatric Medicine, Beijing Tongren Hospital, Capital Medical University, Beijing, China; ^6^Department of Psychiatry, The Melbourne Clinic and St Vincent’s Hospital, University of Melbourne, Richmond, VIC, Australia; ^7^Division of Psychiatry, School of Medicine, University of Western Australia, Perth, WA, Australia; ^8^University of Notre Dame Australia, Fremantle, WA, Australia

**Keywords:** depression, empty-nest, elderly, China, meta-analysis

## Abstract

**Background:**

Depressive symptoms are common in empty-nest elderly in China, but the reported prevalence rates across studies are mixed. This is a meta-analysis of the pooled prevalence of depressive symptoms (depression hereafter) in empty-nest elderly in China.

**Methods:**

Two investigators independently conducted a systematic literature search in both English (PubMed, EMBASE, PsycINFO, Web of Science, and Cochrane Library) and Chinese (CNKI and Wan Fang) databases. Data were analyzed using the Comprehensive Meta-Analysis program.

**Results:**

A total of 46 studies with 36,791 subjects were included. The pooled prevalence of depression was 38.6% (95%CI: 31.5–46.3%). Compared with non-empty-nest elderly, empty-nest elderly were more likely to suffer from depression (OR=2.0, 95%CI: 1.4 to 2.8, *P*<0.001). Subgroup and meta-regression analyses revealed that mild depression were more common in empty-nest elderly than moderate or severe depression (*P*<0.001). In addition, living alone (*P*=0.002), higher male proportion (*β*=0.04, *P*<0.001), later year of publication (*β*=0.09, *P*<0.001) and higher study quality score (*β*=0.62, *P*<0.001) were significantly associated with higher prevalence of depression.

**Conclusion:**

In this meta-analysis, the prevalence of depression in empty-nest elderly was high in China. Considering the negative impact of depression on health outcomes and well-being, regular screening and appropriate interventions need to be delivered for this vulnerable segment of the population.

## Introduction

Empty-nest elderly refers to older adults who have no children or whose children have already left home and thus live alone or with their spouse or older parents (1, 2). China has the world's largest elderly population. The China Statistical Yearbook reported that adults aged 60 and above accounted for 17.9% of the total population by the end of 2018 (3). It was estimated that the total number of empty-nest elderly in China will reach 118 million by 2020 (4), and the proportion of families with empty-nest elderly will reach 90% of all families in China by 2030 (5, 6). Due to the poor general health status associated with aging, and inadequate social supports, empty-nest elderly are more likely to suffer from physical, psychological, and social problems (7, 8).

Compared to their younger counterparts, older adults are usually at a higher risk of developing psychiatric problems, such as depressive symptoms (depression hereafter), which is associated with a range of negative health outcomes, including low quality of life, cognitive decline and even suicide (9, 10). Studies found that the prevalence of depression in empty-nest elderly was significantly higher than those living with children (11–13). In order to reduce the negative impact of depression on health outcome and daily life, understanding the epidemiology of depression in empty-nest elderly and its associated factors are important to develop preventive measures and allocate health resources. Previous studies on the prevalence of depression have reported mixed findings (11, 14). A meta-analysis of 18 studies found that the prevalence of depression in Chinese empty-nest elderly was 40.4% (15). However, there were several limitations of the Xin et al. study, such as the inclusion of only two international databases (PubMed and Science Direct), short time period (2000 to 2012) and only inclusion of studies using the Geriatric Depression Scale (GDS) or the Self-rating Depression Scale (SDS). Therefore, an unknown number of studies on epidemiology of depression in this population were likely to be omitted from this previous meta-analysis. As Xin et al's study was published in Chinese language, it is mostly inaccessible to international readerships. Furthermore, quality assessment of the included studies and certain sophisticated analyses, such as meta-regression and sensitivity analyses, were not conducted.

In the past years, more than 20 studies on prevalence of depression in Chinese empty-nest elderly have been published, which gave us the impetus to conduct an updated meta-analysis with an adequate statistical power and perform sophisticated analyses including meta-regression and sensitivity analyses. In addition, apart from the GDS and SDS, other measures, such as the Patient Health Questionnaire (PHQ), the Hamilton Depression Scale (HAMD), and the Hospital Anxiety and Depression Scale (HAD), were also used in the newly published studies. Therefore, an updated systematic review and meta-analysis of epidemiological surveys were conducted to estimate the prevalence of depression in Chinese empty-nest elderly.

## Methods

### Data Sources and Search Strategy

This meta-analysis was conducted following the Preferred Reporting Items for Systematic Review and Meta-Analyses (PRISMA) principle (16). The research protocol was registered with the International Prospective Register of Systematic Reviews (PROSPERO: CRD42020168782). Relevant publications were independently searched by two investigators (HHZ and YYJ) in both international (PubMed, EMBASE, Web of Science, PsycINFO and Cochrane Library) and Chinese (China National Knowledge Infrastructure and Wan Fang) databases, from inception dates of the target databases to January 1, 2020 using the following search words: depressi*, epidemiology, prevalence, rate, percentage, old*, elderly, aged, aging, China, and Chinese. Additional articles were searched manually in the reference lists of included studies and reviews (15, 17, 18). In case that multiple papers were published based on a single dataset, the one with the largest sample size was included. First or corresponding authors of included studies were contacted for additional information if necessary.

### Inclusion and Exclusion Criteria

Inclusion criteria were established following the PICOS acronym: Participants (P): empty-nest elderly; Intervention (I): not applicable; Comparison (C): not applicable in epidemiological surveys or non-empty-nest elderly in comparative studies; Outcomes (O): prevalence and severity of depression; Study design (S): cross-sectional or comparative studies conducted in mainland China (China thereafter) published in English- or Chinese-language journals reporting prevalence and/or severity of depression measured by standardized assessment scales, such as the GDS, Geriatric Mental State Schedule (GMS), SDS and others. Studies conducted in special populations (e.g., hospitalized patients) were excluded. The primary outcome measure was prevalence of depression.

### Study Selection, Data Extraction and Quality Assessment

The same two investigators independently screened relevant publications by reading titles and abstracts and then the full texts for eligibility. They independently extracted the following participation and study characteristics: survey period, study site, year of publication, sampling method, sample size, mean age, response rate, scales on depression and their cut-off values. Any disagreement in literature search and data extraction was resolved by consensus between the investigators, or a discussion with a senior investigator.

Following previous studies (19, 20), study quality was assessed using the Parker's instrument for epidemiological studies (21) which covers the following domains: the targeted population was defined clearly; complete, random or consecutive recruitment was used; response rate was equal or more than 70%; representativeness of sample was demonstrated or justified; defined diagnostic criteria was used; validated instruments for diagnosis was used. The total score ranged from 0 to 6, with higher scores indicating better study quality.

### Statistical Analysis

The Comprehensive Meta-Analysis software, Version 2.0 (CMA 2.0) was used to analyze data (http://www.meta-analysis.com/). Due to different demographic and clinical characteristics between studies, the pooled prevalence of depression and odds ratio (OR) with their 95% confidence intervals (CIs) were calculated using the random-effects model. Sensitivity analyses were conducted by removing each study one by one and then recalculating the prevalence to test the robustness of the primary results. *I^2^* statistic was used to assess heterogeneity between studies, with *I^2^* of >50% indicating high heterogeneity (22). Subgroup analyses were conducted to examine the sources of heterogeneity based on the following categorical variables: age (60–79 vs. ≥80 years), marital status (married vs. unmarried), living arrangement (living alone vs. others), living area (rural vs. urban), education level (primary and below vs. secondary and above), publication language (Chinese vs. English), study site (multicenter vs. single site), economic region (eastern vs. other regions), sampling method (random sampling vs. others), assessment instrument of depression (GDS or GMS vs. others), severity of depression (mild vs. moderate/severe), and sample size (<362 vs. ≥362 using median splitting method). The following continuous variables were analyzed with meta-regression analyses as potential sources of heterogeneity if there were more than 10 included studies: proportion of males, quality assessment score, and year of publication. Publication bias was assessed using funnel plots and Egger's test (23). Significance level was set at 0.05 (two-sided) in all analyses.

## Results

### Search Results and Study Characteristics

**Figure 1:**
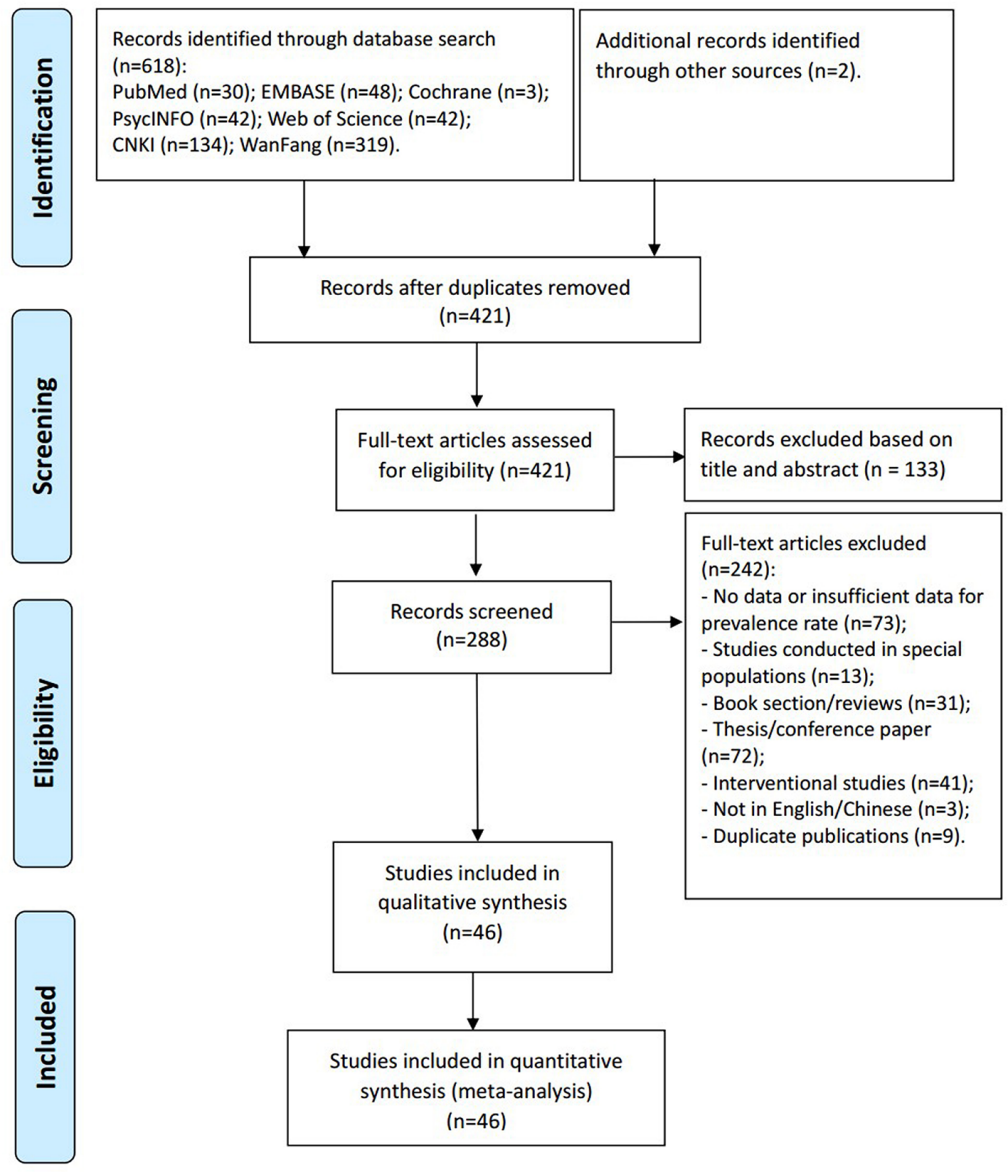
PRISMA flow chart.

Of the 618 papers identified in the literature search and 2 papers identified through other sources, 46 studies with 36,791 participants met study inclusion criteria and were included (**Figure 1**). Eight studies were published in English- and 38 in Chinese-language journals. The sample size ranged from 50 to 5,289 subjects. Thirty studies used the GDS, 10 used the SDS, 2 used the GMS, 2 used the Patient Health Questionnaire (PHQ-9), one used HAMD and one used the HAD. All were cross-sectional studies. Study quality scores ranged between 3 and 6 (**Table 1**).

**Table 1:** Characteristic of studies included in this meta-analysis.

No.	Studies	Publication language	Study location	Sampling method	Participants			Prevalence of depression			Severity of depression		References	Quality assessment score
					Sample size	M%	Mean age (Mean ± SD)	Assessment scale	Cut-off	Events	Mild	Moderate/severe events		
1	Bi and Wu, 2016	C	Shandong	NR	198	44.9	NR	SDS	NR	33	NR	NR	(24)	5
2	Cao, et al., 2012	C	Jilin	Random	454	51.3	NR	SDS	≥50	251	178	73	(25)	6
3	Chang, et al., 2016	E	Liaoning	Random Stratifed Cluster	1,830	54.1	66.97(5.45)	PHQ-9	≥5	485	365	120	(5)	6
4	Chen and Chu, 2012	C	National	Convenience	1,456	39.4	67.3(2.3)	GDS-30	≥11	697	519	178	(26)	5
5	Cheng, et al., 2015	E	Anhui	Random Stratifed Cluster	381	49.9	69.07(NR)	GDS-30	≥11	109	NR	NR	(27)	6
6	Ding, et al., 2019	C	Anhui	Cluster	660	53.2	72.47(5.64)	SDS	≥50	153	NR	NR	(28)	6
7	Du, et al., 2015	C	Shandong	Convenience	802	39.8	73.28(8.05)	GDS-30	NR	415	316	99	(29)	5
8	Gao, et al., 2014	C	Shandong	Random Stratifed Cluster	82	57.3	NR	GDS-30	≥11	43	38	5	(30)	5
9	Gao, et al., 2017	C	Shandong	Random Cluster	653	45.3	NR	GDS-30	≥11	619	412	207	(11)	6
10	Gong, et al., 2018	E	Anhui	Random Stratifed Cluster	2,486	43.8	NR	GDS-15	≥6	599	NR	NR	(31)	5
11	Hu, et al., 2018	C	Hubei	Random	1,852	48.0	NR	GDS-30	≥11	438	283	155	(32)	5
12	Jia, CK., et al., 2007	C	Hunan	Random Stratifed	328	47.6	70.3(8.7)	GDS-30	≥11	78	58	20	(33)	6
13	Jia, SM., et al., 2007	C	Shanghai	Convenience	229	43.7	NR	GDS-15	≥8	35	NR	NR	(34)	5
14	Li, et al., 2011	C	Guangdong	Cluster	111	NR	NR	SDS	≥50	50	14	36	(35)	5
15	Li, et al., 2013	C	Anhui	Random Cluster	343	53.1	71.22(5.46)	SDS	≥16	93	NR	NR	(36)	5
16	Li, et al., 2014	C	Gansu	Random	200	49.0	70.6(6.52)	GDS-30	≥11	48	45	3	(37)	6
17	Li, et al., 2015	C	Shandong	Random Stratifed Cluster	443	32.7	72.55(7.25)	GDS-15	≥8	88	NR	NR	(38)	5
18	Liang, et al., 2014	C	Xinjiang	Random Stratifed	187	47.6	NR	GDS-30	≥11	149	135	14	(39)	6
19	Liu, et al., 2013	C	Hunan	Stratifed	212	41.0	70.15(7.2)	GDS-30	≥11	44	NR	NR	(40)	6
20	Lu, et al., 2019	E	Shanxi	Random Stratifed Cluster	1,593	44.4	NR	SDS	≥50	774	465	309	(41)	6
21	Ma, et al., 2012	C	National	Random Cluster	1,760	46.1	70.82(6.95)	GMS	≥1	144	NR	NR	(14)	6
22	Pan and Wang, 2012	C	Chongqing	Random Stratifed	500	49.6	NR	GDS-30	NR	467	400	67	(13)	6
23	Shen, et al., 2012	C	Hebei	Random Stratifed	1,785	47.5	72 (9)	GDS-30	≥11	353	258	95	(42)	6
24	Shi, et al., 2009	C	Shandong	NR	152	57.2	72.4(7.15)	HAMD	>20	54	NR	NR	(43)	5
25	Su, et al., 2012	E	Hunan	Random Cluster	809	51.5	70.09(7.9)	GDS-30	≥11	593	512	81	(6)	6
26	Su, et al., 2016	C	Guangdong	Cluster	1,035	48.0	69.34(6.26)	GDS-30	≥11	168	139	29	(44)	5
27	Wang and Wang, 2013	C	Beijing	Convenience	100	49.0	72.6(9.2)	GDS-30	≥11	49	39	10	(45)	5
28	Wang and Wang, 2014	C	Sichuan	Random Stratifed	225	54.7	70.06(6.7)	GDS-30	≥11	113	NR	NR	(46)	6
29	Wang, et al., 2014	C	Shanghai	Convenience	212	45.3	NR	GDS-30	≥11	66	60	6	(47)	5
30	Wang, et al., 2018	C	Shanxi	Convenience	504	43.3	NR	GDS-30	≥11	230	182	48	(48)	5
31	Wu, et al, 2013	C	Gansu	Random	87	58.6	NR	SDS	>50	73	NR	NR	(12)	4
32	Xia, et al., 2010	C	Jilin	NR	50	54.0	NR	GDS-30	≥11	26	18	8	(49)	3
33	Xie and Gao, 2009	C	Jilin	Random	279	39.4	NR	GDS-15	≥8	42	NR	NR	(50)	6
34	Xie, et al., 2009	C	Hunan	Convenience	459	53.2	69.52(7.51)	GDS-30	≥11	371	336	35	(51)	5
35	Xie, et al., 2010	E	Hunan	Random Cluster	231	53.2	69.53(7.53)	GDS-30	≥11	184	167	17	(52)	6
36	Xu, 2010	C	Shanghai	Cluster	1,091	51.2	NR	SDS	≥50	118	NR	NR	(53)	6
37	Xu, 2017	C	Jiangsu	Random	276	47.1	NR	GDS-30	≥11	99	85	14	(54)	6
38	Xu, et al., 2015	C	NR	NR	186	44.1	71.6(NR)	SDS	≥50	55	NR	NR	(55)	5
39	Zeng, et al., 2018	C	Zhejiang	Random Stratifed Cluster	162	56.8	73.25(2.58)	GDS-15	≥8	114	NR	NR	(56)	6
40	Zhai, et al., 2015	E	Zhejiang	Random	5,289	48.4	NR	PHQ-9	≥5	613	NR	NR	(57)	5
41	Zhang and Zhang, 2018	C	Shanxi	Random Stratifed Cluster	335	46.0	68.9(7.26)	GDS-15	≥5	107	NR	NR	(58)	6
42	Zhang, et al., 2010	C	Yunnan	Random	199	47.7	NR	GDS-30	≥11	46	NR	NR	(59)	6
43	Zhang, et al., 2016	C	National	Convenience	203	47.3	69.92(6.92)	GDS-30	≥11	106	75	31	(60)	5
44	Zhang, et al., 2019	E	Shanxi	Random Cluster	4,901	51.9	68.5(2.5)	SDS	≥50	3,147	1,776	1,371	(61)	6
45	Zhou, et al., 2008	C	Anhui	Cluster	861	51.2	NR	GMS	≥1	83	NR	NR	(62)	6
46	Zhou, et al., 2009	C	Shanghai	Random Stratifed	600	31.2	72.6(6.7)	HAD	≥8	93	NR	NR	(63)	5

C, Chinese; E, English; NR, Not Reported; M%, Male percent; GDS, Geriatric Depression Scale; GMS, Geriatric Mental State Schedule; SDS, Self-rating depression scale; PHQ, Patient Health Questionnaire; HAMD, Hamilton Depression Scale; HAD, Hospital Anxiety and Depression Scale.

### Prevalence of Depression in Empty-Nest Elderly

**Figure 2:**
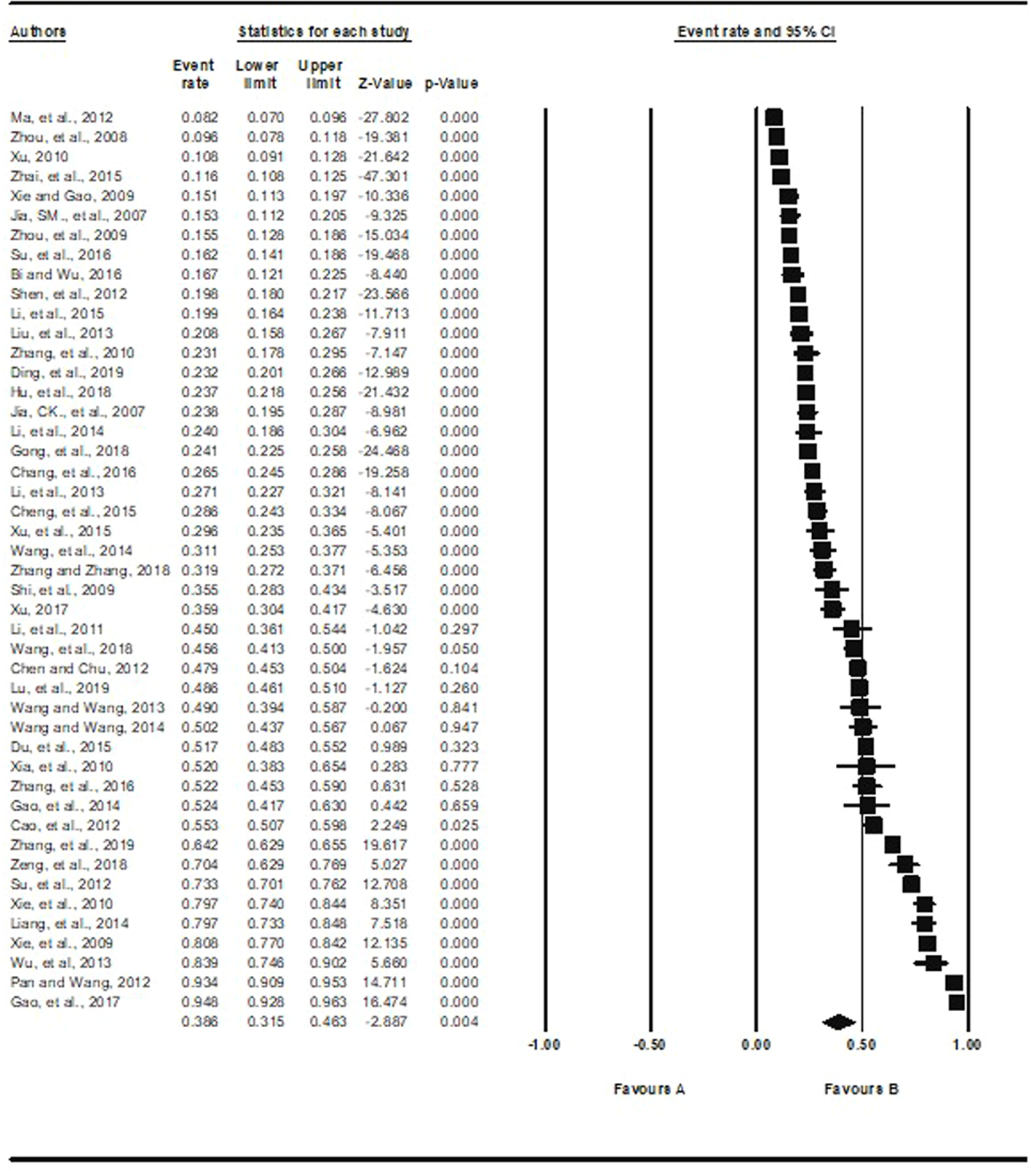
Prevalence of depression in empty-nest elderly.

Based on the 46 studies with available data, the pooled prevalence of depression was 38.6% (95%CI: 31.5% to 46.3%, *I^2^* = 99.3%), ranging from 8.2% (95%CI: 7.0% to 9.6%) (14) to 94.8% (95%CI: 92.8% to 96.3%) (11) (**Figure 2**).

### Subgroup and Meta-Regression Analyses

**Table 2** shows the results of the subgroup analyses. Mild depression were more common in empty-nest elderly than moderate or severe depression (*P*<0.001). Living alone was associated with higher prevalence of depression (*P*=0.002). Age, marital status, education level, living in urban or rural, publication language, study site, economic region, sampling method, sample size, and assessment scale did not have moderating effects on the prevalence of depression. Meta-regression analyses revealed that higher prevalence of depression was significantly associated with higher proportion of males (*β*=0.04, *P*<0.001) (**Supplementary Figure 3**), later year of publication (*β*=0.09, *P*<0.001) (**Supplementary Figure 4**), and higher study quality score (*β*=0.62, *P*<0.001) (**Supplementary Figure 5**).

**Table 2:** Subgroup analyses of prevalence of depressive symptoms in empty-nest elderly.

Subgroups	Categories (number of studies)	Effect size (%)	95% CI		Events	Sample size	*I^2^*	*P* (within subgroup)	*P (*across subgroups)
**Publication language**	Chinese (n=38)	37.7	30.2	45.8	6,211	19,271	98.86	<0.001	0.25 (0.62)
	English (n=8)	43.1	25.1	63.0	6,504	17,520	99.80	<0.001	
**Study site**	Multicenter (n=37)	39.2	31.3	47.6	11,596	32,860	99.40	<0.001	0.07 (0.80)
	Single site (n=9)	36.4	20.3	56.3	1,119	3,931	98.79	<0.001	
**Economic region**	East (n=17)	33.1	22.9	45.0	3,010	13,220	99.05	<0.001	1.57 (0.21)
	Other areas (n=28)	42.4	33.7	51.7	9,650	23,385	99.32	<0.001	
**Sampling method**	Random (n=28)	42.6	32.6	53.2	9,962	28,270	99.52	<0.001	1.42 (0.23)
	Others (n=14)	33.0	22.5	45.4	2,585	7,935	98.95	<0.001	
**Definition of elderly by age**	≥60 (n=41)	36.3	29.0	44.4	12,039	35,292	99.39	<0.001	0.09 (0.76)
	Other definition (n=3)	40.2	19.9	64.5	454	1,225	98.28	<0.001	
**Sample size****^a^**	<362 (n=23)	39.9	31.7	48.8	1,707	4,587	96.62	<0.001	0.14 (0.71)
	≥362 (n=23)	37.3	27.3	48.5	11,008	32,204	99.65	<0.001	
**Assessment scale**	GDS or GMS (n=32)	41.3	32.6	50.5	6,723	19,296	99.10	<0.001	0.97 (0.33)
	Other scale (n=14)	32.8	20.8	47.6	5,992	17,495	99.62	<0.001	
**Living area**	Rural (n=4)	36.6	15.7	64.1	2,465	4,492	99.44	<0.001	0.12 (0.73)
	Urban (n=4)	30.3	12.3	57.3	1,315	3,039	99.29	<0.001	
**Marital status**	Married (n=11)	26.6	16.2	40.6	3,365	9,022	99.31	<0.001	3.51 (0.06)
	Others^b^ (n=11)	44.6	31.9	58.1	1,853	3,566	97.83	<0.001	
**Age**	80 years and above (n=10)	33.3	19.1	51.5	576	1,343	96.51	<0.001	0.23 (0.63)
	60-79 years (n=10)	27.8	15.2	45.3	3,925	10,326	99.54	<0.001	
**Living arrangement**	Live alone (n=12)	39.2	31.1	47.9	1,101	3,092	94.36	<0.001	**9.61 (0.002)**
	Not alone (n=12)	22.6	17.1	29.4	2,002	9,117	97.63	<0.001	
**Education level**	Primary and below (n=9)	30.8	15.7	51.4	3,505	7,839	99.48	<0.001	0.45 (0.50)
	Secondary and above (n=9)	24.0	15.9	34.5	892	2,965	96.77	<0.001	
**Severity of depressive symptoms**	Mild (n=25)	37.4	30.2	45.1	6,875	20,613	98.95	<0.001	**53.38 (<0.001)**
	Moderate or severe (n=25)	9.8	7.3	13.1	3,031	20,613	98.06	<0.001	

a Dichotomized using the median split method. ^b^Never married, widowed, divorced, or separated. Bolded values: P<0.05.

GDS, Geriatric Depression Scale; GMS, Geriatric Mental State Schedule.

### Prevalence of Depression in Empty-Nest and Non-Empty-Nest Elderly

Based on 19 comparative studies (16,041 empty-nest and 13,203 non-empty-nest participants) with available data, the prevalence of depression was 44.2% (95%CI: 30.9% to 54.4%, *I^2^* = 99.2%) in the empty-nest, and 26.3% (95%CI: 18.3% to 36.3%, *I^2^* = 98.8%) in the non-empty-nest groups. Compared with non-empty-nest elderly, empty-nest elderly were more likely to suffer from depression (OR=2.0, 95%CI: 1.4 to 2.8, *I^2^* = 94.9%, *P*<0.001) (**Supplementary Figure 1**).

### Sensitivity Analysis and Publication Bias

**Figure 3:**
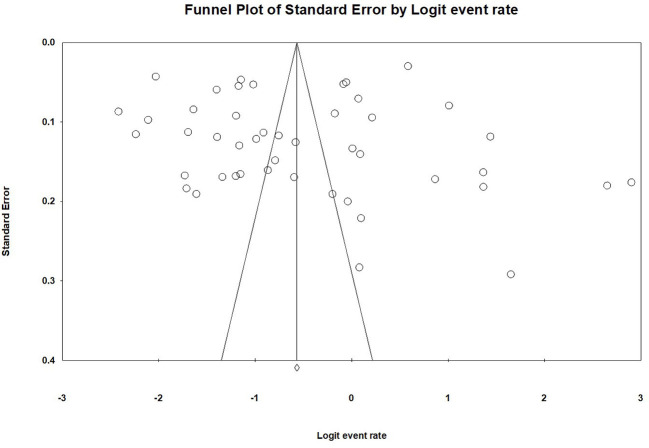
Publication bias of the 46 included studies reporting prevalence of depression in empty-nest elderly.

When each study was excluded sequentially, the primary results did not change significantly, indicating that there was no outlying study that could significantly influence robustness of the primary results. No publication bias was found in the 46 studies on the prevalence of depression according to visual funnel plot (**Figure 3**) and Egger's test (t=0.08, 95%CI: −7.39 to 6.84; *P*=0.94). **Supplementary Figure 2** shows the funnel plot of the 19 comparative studies. Egger's test (t=1.76, 95%CI: -0.71 to 7.81; *P*=0.10) also did not show publication bias.

## Discussion

This updated systematic review and meta-analysis found that the pooled prevalence of depression in Chinese empty-nest elderly was 38.6% (95%CI: 31.5–46.3%), which is significantly higher than the corresponding figure in general older population in China (22.6%; 95%CI: 18.9–26.7%) (64) and in Western countries (19.5%; 95%CI: 19.1–19.8%) (65). In addition, this was the first meta-analysis found that empty-nest elderly were more likely to have depression than their non-empty-nest counterparts (OR=2.0). In China, there are around 118 million empty-nest elderly (4), which, based on the results of this study, translates to approximately 37.17–54.63 million elderly suffering from depression.

With the rapid economic expansion in China, in recent decades growing number of young people have left their hometowns to seek employment elsewhere, thus leaving their parents to live by themselves at home. However, social support systems for the elderly have not been well established, particularly in many rural areas (66, 67). Not surprisingly, compared to those living with children, empty-nest elderly have poorer physical and mental health, greater dissatisfaction with their health and income (57), poorer family function (68, 69), lower quality of life (70, 71), poorer sleep quality (72), and more loneliness (73, 74), all of which may increase the risk of depression. The prevalence of depression in the population of empty-nest elderly in this meta-analysis (38.6%; 95%CI: 31.5–46.3%) is lower than previous findings (40.4%; 95%CI: 28.6–52.2%) (15), although the difference did not reach significant level. With 28 more studies and a larger sample size (36,791 vs. 4,855), the findings of the current study are more reliable than those of Xin et al's due to increased statistical power. In addition, the prevalence of depression between empty-nest elderly and their non-empty-nest counterparts was compared in the current, but not in the Xin et al.'s meta-analysis. Furthermore, Xin et al. found that economic region, living area, assessment scale and sampling method were significantly associated with the prevalence of depression in empty-nest elderly, all of which however have were not confirmed in this meta-analysis. The Xin et al.'s meta-analysis only included 18 studies. Moreover, meta-regression analyses were performed in this meta-analysis, but not in the Xin et al. study.

Mild depression was more common than moderate and severe depression in empty-nest elderly. Moderate and severe depression is associated with substantial distress and functional impairment, and therefore it is likely to receive appropriate clinical attention. In contrast, empty-nest elderly with mild depression are less likely to seek help from mental health services (75, 76), which could explain why they have more common mild depression. The association between gender and depression in the elderly is unclear. Some studies found that female elderly are more likely to suffer from depression (15, 64), but this was not found in other studies (77, 78). In this meta-analysis, prevalence of depression was higher in male elderly. Chinese female elderly usually prefer to participate in group activities, such as outdoor square dance or Tai Chi, which could reduce the likelihood of loneliness and the risk of depression (79–81). Higher prevalence of depression was associated with better study quality. In high quality studies interviewers are usually well trained on the use of assessment instruments, therefore, depression is more likely to be identified.

Living alone can be associated with financial hardship, more frequent medical conditions, widowhood, and poor social support (14, 52, 74, 82). Further, there is an increasing trend recently that the younger Chinese generation have infrequently visited their parents living in their hometowns due to their busier and more demanding lives working in cities (70). All these factors could explain the association between higher prevalence of depression and living alone and more recently published studies.

The strengths of this meta-analysis are the inclusion of recently published studies, the large sample size, large number of included studies both from English and Chinese databases, and the application of sophisticated analyses (sensitivity, subgroup and meta-regression analyses). However, several limitations need to be noted. First, the included studies were conducted in 22 out of the 34 provinces/municipalities/autonomous regions in China, limiting the generalizability of the findings. Second, there was heterogeneity between studies, which is inevitable in meta-analysis of epidemiological studies (83, 84), although subgroup analyses have been conducted to address this shortcoming. Heterogeneity was probably due to different demographic characteristics, inclusion/exclusion criteria, and sampling methods employed by the included studies. In addition, pertinent demographic and clinical factors associated with depression, such as social support and physical comorbidities, were not examined due to insufficient data. Third, there were only a small number of studies available in subgroup analysis when comparing educational level (n=9) and living area (n=4) reducing the statistical power of the findings.

In conclusion, this meta-analysis found that the prevalence of depression in empty-nest elderly in China was high and significantly greater than non-empty-nest older people. Considering the negative impact of depression on health outcomes and well-being, regular screening and appropriate interventions are needed for this vulnerable segment of the population.

## Data Availability Statement

All datasets presented in this study are included in the article/**Supplementary Material**.

## Author Contributions

Study Design: Y-TX. Data collection, analysis and interpretation of data: H-HZ, Y-YJ, W-WR, Q-EZ. Drafting of the manuscript: H-HZ, M-ZQ, Y-TX. Critical revision of the manuscript: CN, GU. All authors contributed to the article and approved the submitted version.

## Funding

The study was supported by the National Science and Technology Major Project for investigational new drug (2018ZX09201-014), the Beijing Municipal Science & Technology Commission (No. Z181100001518005), and the University of Macau (MYRG2019-00066-FHS).

## Conflict of Interest

The authors declare that the research was conducted in the absence of any commercial or financial relationships that could be construed as a potential conflict of interest.
