# Supplementary material for: Prevalence of Depression Among Empty-Nest Elderly in China: A Meta-Analysis of Observational Studies
Source: Front Psychiatry. 2020 Jul 7;11:608. doi: 10.3389/fpsyt.2020.00608 (PMC7358371; doi:10.3389/fpsyt.2020.00608)
Supplement: Supplementary file 1 [file DataSheet_1.docx]

**Supplementary Table 1.** **Quality assessment of studies included in this meta-analysis.**

| **Study** | **1.The targeted population was defined clearly** | **2.** **Complete, random or consecutive recruitment** | **3. Response rate was equal or more than 70%** | **4. Representativeness of sample** | **5.** **Use of defined diagnostic criteria for depression** | **6. Use of validated instruments for depression** | **Total** |
| --- | --- | --- | --- | --- | --- | --- | --- |
| Bi and Wu, 2016 | 1 | 0 | 1 | 1 | 1 | 1 | 5 |
| Cao, et al., 2012 | 1 | 1 | 1 | 1 | 1 | 1 | 6 |
| Chang, et al., 2016 | 1 | 1 | 1 | 1 | 1 | 1 | 6 |
| Chen and Chu, 2012 | 1 | 0 | 1 | 1 | 1 | 1 | 5 |
| Cheng, et al., 2015 | 1 | 1 | 1 | 1 | 1 | 1 | 6 |
| Ding, et al., 2019 | 1 | 1 | 1 | 1 | 1 | 1 | 6 |
| Du, et al., 2015 | 1 | 0 | 1 | 1 | 1 | 1 | 5 |
| Gao, L., et al., 2014 | 1 | 1 | 1 | 0 | 1 | 1 | 5 |
| Gao, et al., 2017 | 1 | 1 | 1 | 1 | 1 | 1 | 6 |
| Gong, et al., 2018 | 1 | 1 | 0 | 1 | 1 | 1 | 5 |
| Hu, et al., 2018 | 1 | 1 | 0 | 1 | 1 | 1 | 5 |
| Jia, CK., et al., 2007 | 1 | 1 | 1 | 1 | 1 | 1 | 6 |
| Jia, SM., et al., 2007 | 1 | 0 | 1 | 1 | 1 | 1 | 5 |
| Li, et al., 2011 | 1 | 1 | 0 | 1 | 1 | 1 | 5 |
| Li, et al., 2013 | 1 | 1 | 0 | 1 | 1 | 1 | 5 |
| Li, F., et al., 2014 | 1 | 1 | 1 | 1 | 1 | 1 | 6 |
| Li, et al., 2015 | 1 | 1 | 0 | 1 | 1 | 1 | 5 |
| Liang, et al., 2014 | 1 | 1 | 1 | 1 | 1 | 1 | 6 |
| Liu, et al., 2013 | 1 | 1 | 1 | 1 | 1 | 1 | 6 |
| Lu, et al., 2019 | 1 | 1 | 1 | 1 | 1 | 1 | 6 |
| Ma, et al., 2012 | 1 | 1 | 1 | 1 | 1 | 1 | 6 |
| Pan and Wang, 2012 | 1 | 1 | 1 | 1 | 1 | 1 | 6 |
| Shen, et al., 2012 | 1 | 1 | 1 | 1 | 1 | 1 | 6 |
| Shi, et al., 2009 | 1 | 0 | 1 | 1 | 1 | 1 | 5 |
| Su, et al., 2012 | 1 | 1 | 1 | 1 | 1 | 1 | 6 |
| Su, et al., 2016 | 1 | 1 | 0 | 1 | 1 | 1 | 5 |
| Wang and Wang, 2013 | 1 | 0 | 1 | 1 | 1 | 1 | 5 |
| Wang and Wang, 2014 | 1 | 1 | 1 | 1 | 1 | 1 | 6 |
| Wang, et al., 2014 | 1 | 0 | 1 | 1 | 1 | 1 | 5 |
| Wang, et al., 2018 | 1 | 0 | 1 | 1 | 1 | 1 | 5 |
| Wu, et al, 2013 | 0 | 1 | 1 | 0 | 1 | 1 | 4 |
| Xia, et al., 2010 | 1 | 0 | 0 | 0 | 1 | 1 | 3 |
| Xie and Gao, 2009 | 1 | 1 | 1 | 1 | 1 | 1 | 6 |
| Xie, et al., 2009 | 1 | 0 | 1 | 1 | 1 | 1 | 5 |
| Xie, et al., 2010 | 1 | 1 | 1 | 1 | 1 | 1 | 6 |
| Xu, 2010 | 1 | 1 | 1 | 1 | 1 | 1 | 6 |
| Xu, 2017 | 1 | 1 | 1 | 1 | 1 | 1 | 6 |
| Xu, et al., 2015 | 1 | 0 | 1 | 1 | 1 | 1 | 5 |
| Zeng, et al., 2018 | 1 | 1 | 1 | 1 | 1 | 1 | 6 |
| Zhai, et al., 2015 | 1 | 1 | 0 | 1 | 1 | 1 | 5 |
| Zhang and Zhang, 2018 | 1 | 1 | 1 | 1 | 1 | 1 | 6 |
| Zhang, et al., 2010 | 1 | 1 | 1 | 1 | 1 | 1 | 6 |
| Zhang, et al., 2016 | 1 | 0 | 1 | 1 | 1 | 1 | 5 |
| Zhang, et al., 2019 | 1 | 1 | 1 | 1 | 1 | 1 | 6 |
| Zhou, et al., 2008 | 1 | 1 | 1 | 1 | 1 | 1 | 6 |
| Zhou, et al., 2009 | 1 | 1 | 0 | 1 | 1 | 1 | 5 |

**Supplementary Figure 1. Comparison of prevalence of depressive symptoms between empty-nest and non-empty-nest elderly.**

**Supplementary Figure 2. Publication bias of 19 studies comparing prevalence of depressive symptoms between empty-nest and non-empty-nest groups.**

**Supplementary Figure 3. Meta regression of male proportion on prevalence of depressive symptoms in empty-nest elderly.**

Note: Slope=0.044, Intercept=-2.653, P<0.001.

**Supplementary Figure 4. Meta regression of publication year on prevalence of depressive symptoms in empty-nest elderly.**

Note: Slope=0.087, Intercept=-175.352, P<0.001.

**Supplementary Figure 5. Meta regression of study quality score on prevalence of depressive symptoms in empty-nest elderly.**

Note: Slope=0.623, Intercept=-4.029, P<0.001.
